# Acute Pyogenic Ankle Monoarthritis Due to Tuberculosis: A Real-Life Clinical Conundrum

**DOI:** 10.7759/cureus.94517

**Published:** 2025-10-13

**Authors:** Ozair Ali, Rahim Abbas, Shing F Chau, Puneet Srivastava, Mithun Chakravorty

**Affiliations:** 1 Internal Medicine, Barnsley Hospital NHS Foundation Trust, Barnsley, GBR; 2 Radiology, University Hospitals of Derby and Burton NHS Foundation Trust, Derby, GBR; 3 Rheumatology, Srigyan Rheumatology Clinic, Noida, IND; 4 Rheumatology, Barnsley Hospital NHS Foundation Trust, Barnsley, GBR

**Keywords:** ankle, extrapulmonary tb, infectious arthritis, osteoarticular tb, septic arthritis, tuberculosis

## Abstract

Musculoskeletal tuberculosis (TB) usually involves the spine, hip, or knee. Ankle disease is rare and can present insidiously, which contributes to delayed diagnosis and poor outcomes. We report a case of an 86-year-old Chinese woman who presented with acute pain and swelling of her left ankle for two days, fevers and inability to bear weight. Past medical history included ischemic heart disease, congestive cardiac failure and recurrent urinary tract infections. Examination revealed a fever of 39.5°C and a tender, swollen ankle with restricted movement. Laboratory tests showed anaemia, lymphopenia and elevated C-reactive protein. Ankle X-ray was unremarkable. Joint aspiration yielded pus, but cultures were negative. Despite intravenous antibiotics, her symptoms persisted. MRI demonstrated marked ankle synovitis, and joint washout revealed pus around the ankle and flexor tendons. She subsequently became hypoxic, and chest imaging suggested possible miliary TB. Retrospective analysis of the aspirate confirmed acid-fast bacilli and TB polymerase chain reaction (PCR) positivity. Anti-tuberculous therapy (ATT) was commenced, but the patient deteriorated and died on day 19 of admission. This case highlights the diagnostic challenge of ankle TB, which may present acutely and mimic septic arthritis. Delayed recognition contributes to poor outcomes, and pulmonary involvement can provide important diagnostic clues. TB should be considered in the differential diagnosis of acute monoarthritis, particularly in high-risk individuals. Early recognition and prompt initiation of therapy are critical to improving patient outcomes.

## Introduction

Tuberculosis (TB) is an infectious disease caused by the bacterium *Mycobacterium tuberculosis*. It was historically the world’s leading cause of death from a single infectious agent until coronavirus disease (COVID-19) emerged in 2020. Since 2023, it has overtaken COVID-19, and an estimated 10.8 million people fell ill with TB worldwide as per the World Health Organisation (WHO) report [1]. TB is an endemic disease, particularly in developing countries, but its incidence remains low in England, around 7.75 cases per 100,000 population [2].

TB most commonly affects the lungs and typically presents with symptoms of prolonged cough, chest pain, weight loss, fever and night sweats [1]. When TB occurs in organ systems other than the lungs, it is referred to as extrapulmonary TB (EPTB). EPTB can affect almost any organ system, including the kidneys, brain, spine and skin [1].

Musculoskeletal TB is a less common extrapulmonary manifestation of the disease, making up about 10% of EPTB cases [3]. Due to its rarity and non-specific symptoms, it can be challenging to differentiate it from other inflammatory or degenerative joint disorders, and the diagnosis is often delayed.

We highlight a rare case of isolated ankle TB, which presented as an acute septic joint, and despite initiation of anti-tuberculous treatment, the patient died.

## Case presentation

An 86-year-old woman of Chinese descent, who emigrated from China to the United Kingdom 50 years ago and has since made occasional return visits to China, presented to the emergency department with a two-day history of a hot, swollen and painful left ankle. She was febrile with a temperature of 39.5°C at presentation and was unable to weight-bear due to the ankle pain. There was no trauma to the ankle and no previous joint swelling. She did not have skin psoriasis or symptoms of seronegative spondyloarthropathy. There was no history of TB or known exposure to individuals infected with TB. No other relevant symptoms were found on systemic enquiry. 

The patient’s past medical history included ischemic heart disease, congestive cardiac failure, and recurrent urinary tract infections. She had never been treated with corticosteroids or immunosuppressive drugs.

One month prior to this hospital admission, she received inpatient treatment for a lower respiratory tract infection and a negative COVID-19 test. No acute abnormalities were noted on her chest CT scan. 

Upon examination on the medical ward, vital signs revealed a temperature of 39.0°C, blood pressure of 130/65 mmHg, a heart rate of 90 beats per minute, an oxygen saturation of 98% on room air, and a respiratory rate of 20 breaths per minute. There was bilateral pitting lower leg oedema with increased warmth, swelling and pain over the left ankle. The range of motion in the ankle was very limited due to pain, and no other joints were painful or swollen. The remainder of the physical examination was normal.

**Figure 1:**
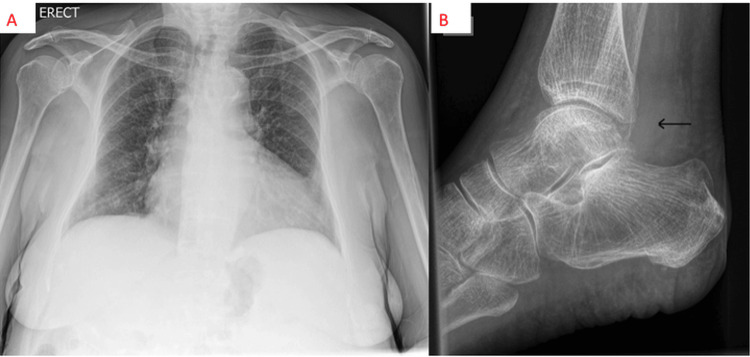
(A) Initial chest X-ray at presentation - lungs appeared clear. (B) Plain radiograph of the left ankle (horizontal beam view). Note the superadded density in the posterior recess of the ankle joint (line arrow), corresponding to synovitis and effusion. No cortical erosion at the time of imaging.

Blood tests revealed mild lymphopenia and chronic anaemia, a raised C-reactive protein (CRP) level of 90 mg/L, and normal renal function with mildly elevated liver function tests. Lactate was 2.0 mmol/L (Table 1). A full septic screen was performed, including blood cultures, chest X-ray (Figure 1) and urinalysis, and these were unremarkable. Ankle X-ray suggested the presence of an ankle effusion without any bony destruction (Figure 1). Ankle aspiration was performed by the Rheumatology Team and demonstrated pus with 2+ leucocytes on microscopy, but no organism or crystals were seen. An urgent orthopaedic review was requested due to the presence of a pyogenic aspirate.

**Table 1:** Summary of blood results at presentation ALP: alkaline phosphatase; ALT: alanine aminotransferase; GGT: gamma-glutamyl transferase; CRP: C-reactive protein

Laboratory Test (units)	Result	Reference Range
Haemoglobin (g/L)	83	119-149
White cell count (×10⁹/L)	7.47	3.7-10
Lymphocytes (×10⁹/L)	0.24	1.0-3.0
Platelet count (×10⁹/L)	191	150-450
Serum sodium (mmol/L)	125	133-146
Serum potassium (mmol/L)	4.5	3.5-5.3
Urea (mmol/L)	6.6	2.5-7.8
Creatinine (μmol/L)	66	51-96
Total bilirubin (µmol/L)	8	<21
ALP (IU/L)	170	30-130
ALT (IU/L)	58	0-40
GGT (g/L)	72	0-50
Albumin(g/L)	17	35-50
CRP (mg/L)	90	0-10
Lactate (mmol/L)	2	0.5-2.2
Blood cultures	Negative	Not applicable

**Figure 2:**
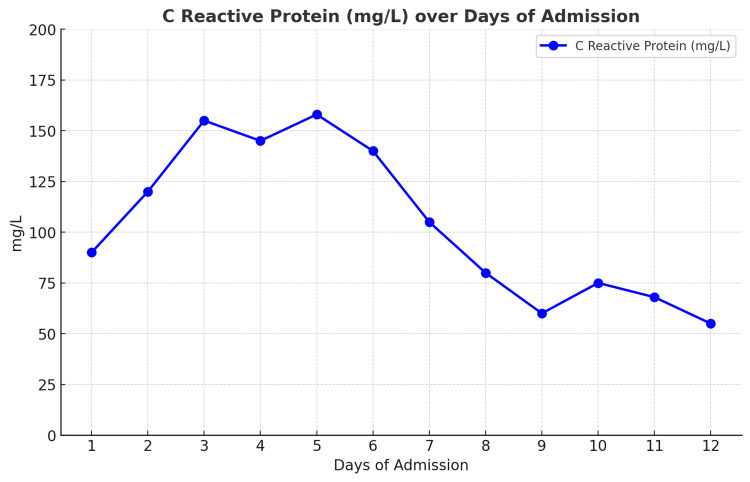
CRP trend through the course of the admission.

**Figure 3:**
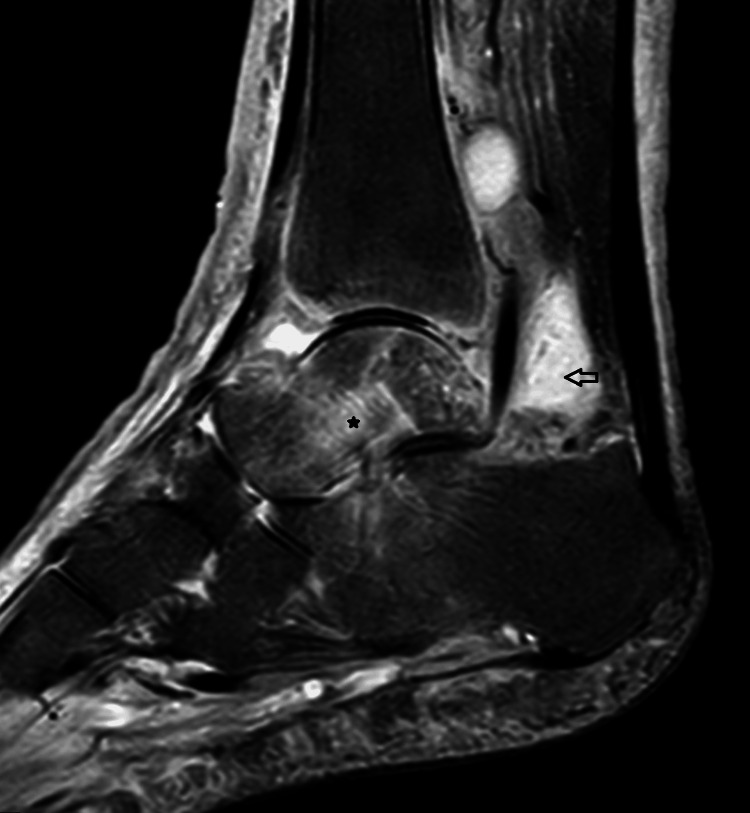
MRI of the left ankle (sagittal STIR sequence). Mixed hyperintense signals within the synovial membrane and joint cavity (blank arrow), indicating the presence of inflammation and increased effusion typical of synovitis, which appears extensive and severe in this case. Reactive marrow oedema is also noted (asterisk).

Intravenous flucloxacillin 2 g four times daily was commenced for suspected septic arthritis; however, the joint aspirate culture was negative. Over the next week, the patient continued to have intermittent fevers, and the left ankle remained painful and swollen. The CRP remained persistently elevated above 100 mg/L (Figure 2) despite broader-spectrum antimicrobial treatment with intravenous piperacillin/tazobactam 4.5 g three times daily, as advised by the Microbiology Team. Multiple sets of blood cultures were negative, and an MRI scan of the ankle showed marked ankle synovitis (Figure 3).

A joint washout was performed by the Orthopaedic Team on day 14 and demonstrated a small amount of pus around the ankle joint and marked pus surrounding the Flexor Digitorum Longus and Flexor Hallucis Longus tendons. Further joint fluid was sent for culture, and this also came back negative.

**Figure 4:**
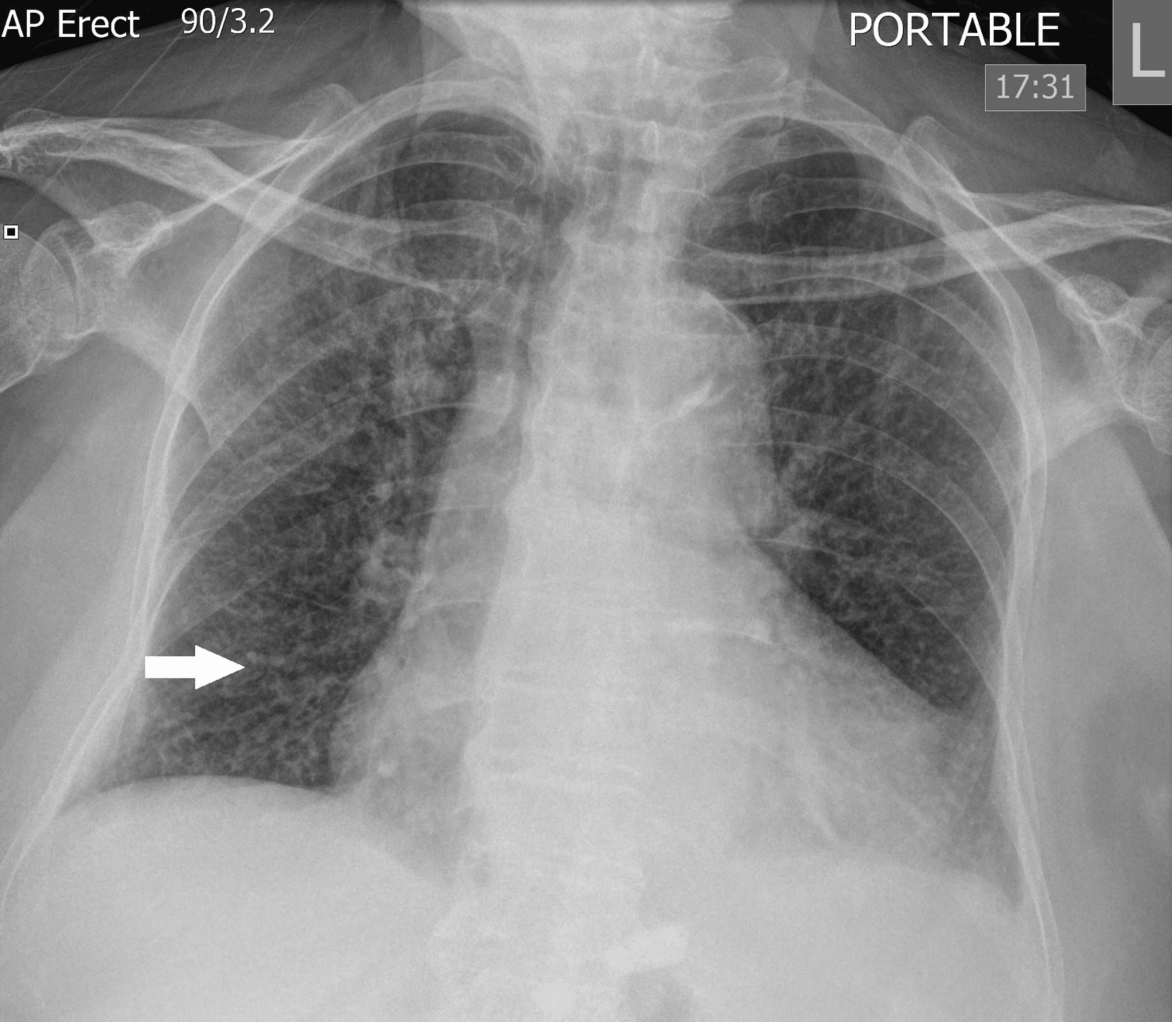
Chest radiograph (AP erect). There are numerous new tiny opacities distributed uniformly in both lungs (white arrow).

Two days after the joint washout, the patient developed shortness of breath with a new oxygen requirement. An urgent chest X-ray suggested numerous small lung infiltrates, which were new compared to the chest X-ray on admission (Figure 4). Miliary TB was suspected, and a retrospective analysis of the joint washout was positive for acid-fast bacilli (AFB), with TB PCR also returning positive. A CT scan of the chest was considered but not performed as the patient was too unwell at the time. A combination of rifampicin, isoniazid and pyrazinamide, along with ethambutol, was commenced on day 16; however, the patient developed severe type 1 respiratory failure and continued to deteriorate despite optimal medical care, and end-of-life care was initiated. The patient died on day 19 of admission.

## Discussion

Musculoskeletal TB is an uncommon manifestation of TB and comprises about 1-3% of all TB cases [3-5]. It has different forms, including spondylitis (Pott’s disease), arthritis and osteomyelitis, which can be acute or subacute in onset. Spinal involvement is the most common, comprising about half of cases of musculoskeletal TB [4]. Tuberculous arthritis is a form of musculoskeletal TB, and the most commonly affected joints are the knee and hip [6]. Involvement of the foot and ankle is rare, and ankle TB is thought to occur in 1-4% of all cases of tuberculous arthritis [5].

Musculoskeletal TB may develop through haematogenous dissemination from a distant primary site, such as the lungs, and pulmonary involvement has been reported to occur concurrently with ankle TB in approximately one-third to one-half of cases [6,7].

Typical clinical manifestations of tuberculous arthritis include a painful, swollen joint with or without loss of joint function. Symptoms are usually of insidious onset over weeks to months. The joint is generally ‘cold’ rather than having typical inflammatory signs such as warmth and erythema [8], and it can be accompanied by constitutional symptoms such as fever and weight loss in about 30% of cases [9]. 

In non-endemic areas, risk factors for developing extrapulmonary TB include patients with immunosuppressive conditions like HIV infection, chronic illnesses such as diabetes mellitus, alcoholism, malignancies, and those receiving corticosteroids or immunosuppressive treatments such as chemotherapy or biologics. Furthermore, local factors such as trauma, surgical interventions, or intravenous drug use may trigger reactivation of TB in adjacent joints [7]. 

Given the low incidence of TB in the UK compared to developing countries, there is often a low index of clinical suspicion, resulting in diagnostic and treatment delays. TB of the foot and ankle can be seen at almost any age [10], and a six-year prospective study evaluating the epidemiology of musculoskeletal TB in Bradford, a large multicultural UK city, found that approximately three-fourths of all patients with musculoskeletal TB were born outside the UK [11].

Radiographic imaging plays a supportive role in the diagnosis of musculoskeletal TB; however, the findings are often non-pathognomonic and non-specific, particularly in the early stages of the disease [12]. The X-ray finding classically associated with tuberculous arthritis is characterised by juxta-articular osteoporosis, peripheral osseous erosions and gradual narrowing of joint space, known as the Phemister triad, but is again non-specific [13]. Some studies have shown that the absence of marrow enhancement on MRI, along with bony erosions, is suggestive of tuberculous arthritis rather than a pyogenic process [14].

The gold standard for diagnosing musculoskeletal TB remains the isolation of Mycobacterium tuberculosis through culture, which also allows for drug susceptibility testing (DST) [15]. Synovial fluid obtained by joint aspiration is commonly subjected to AFB smear, culture and molecular testing such as Nucleic Acid Amplification Test (NAAT) [15,16]. However, AFB smear has low sensitivity in extrapulmonary sites and often fails to detect Mycobacterium TB in joint fluid specimens [17]. Culture positivity in musculoskeletal TB has been reported in the range of 50% to 80%, depending on specimen type, laboratory infrastructure and prior antibiotic exposure [17].

In cases where synovial fluid workup is inconclusive, a synovial biopsy (or bone biopsy) becomes essential. Histopathology typically reveals caseating granulomatous inflammation, sometimes with demonstrable AFB. Biopsy often yields higher diagnostic sensitivity: for example, a recent large-joint study reported histology positivity in approximately 66% of biopsied specimens, and combined culture and histology increases the diagnostic yield [17,18].

Culture-based diagnosis typically requires six to eight weeks or more, depending on laboratory conditions [17,19]. Meanwhile, molecular/NAAT assays (e.g., Xpert MTB/RIF or Ultra) can provide results in hours to days and help guide early management; however, their sensitivity in extrapulmonary specimens is lower than that of culture, and negative results do not reliably exclude disease [16,18,20].

According to the 2016 American Thoracic Society (ATS), Centers for Disease Control and Prevention (CDC) and Infectious Diseases Society of America (IDSA) clinical practice guidelines for the diagnosis of TB in adults and children, NAAT is suggested (conditionally) for specimens from extrapulmonary sites as a supplementary tool to culture, but not as a sole diagnostic method [15,16].

In summary, the diagnostic algorithm in suspected musculoskeletal TB should integrate AFB stain, culture (solid and liquid media), NAAT and biopsy/histopathology, interpreting all findings in the clinical and radiologic context to maximise sensitivity and specificity [15-20].

Treatment of tuberculous arthritis is usually medical in the first instance with anti-tuberculous therapy for a minimum of nine to 12 months [10]. However, joint destruction and draining sinuses can be present in late presentations as well as osteomyelitis [6,7]. Surgical intervention may be necessary for non-healing and severe joint deformities, with procedures such as curettage, debridement, sequestrectomy, bone grafting, fistulectomy, synovectomy, arthrodesis and resection [7,10].

In the majority of reported ankle TB cases, the symptoms evolve slowly, mimicking chronic arthritic conditions such as osteoarthritis or rheumatoid arthritis [7]. Some case series have reported that patients with TB of the foot and ankle had joint symptoms ranging from two months to 1.9 years [10]. It is exceedingly rare to present acutely and mimic a septic monoarthritis as in our case. We have found only one previous case in the literature where this occurred with aspirate-proven TB [7].

The case presented in this report posed a significant diagnostic challenge, particularly given the purulent joint aspirate, which would typically suggest septic arthritis caused by more common bacterial pathogens. The persistent negative fluid and blood cultures, despite ongoing fevers and elevated CRP, were highly unusual. TB was later considered after the patient developed breathlessness and low oxygen saturations, and a repeat chest X-ray showed several small lung infiltrates. Miliary TB was considered, particularly given that the patient’s country of origin had a high prevalence of TB. Also, it was noted that there were calcified intra-abdominal lymph nodes on the CT thorax prior to admission, and this was suggestive of previous TB. There were no other identifiable risk factors for TB (HIV test was negative), and no predisposing conditions for this rare localisation of TB in the ankle joint, e.g., prior trauma or surgery to the ankle. 

In our opinion, the patient likely had pulmonary TB as the initial source that was not detectable on the initial chest X-ray or recent CT thorax, and this caused seeding into the ankle before becoming Miliary. It is possible that a repeat CT scan earlier may have demonstrated miliary TB, but it was not clinically indicated due to lack of respiratory symptoms, normal oxygen saturations, and the low clinical suspicion of TB at the time.

Ziehl-Neelsen (acid-fast) staining and TB PCR facilitated an earlier diagnosis, as TB culture alone would have taken several more weeks.

## Conclusions

Ankle TB is uncommon and can rarely present acutely, mimicking septic arthritis. A high index of clinical suspicion is required, particularly in patients with risk factors for TB. Concomitant pulmonary involvement is relatively common; therefore, chest imaging should be considered. Staining for AFB or TB PCR testing from joint aspirate or synovial biopsy can aid in earlier diagnosis and facilitate prompt initiation of anti-tuberculous therapy. Delaying the diagnosis may lead to catastrophic outcomes.
